# Pulmonary exposure to single-walled carbon nanotubes does not affect the early immune response against *Toxoplasma gondii*

**DOI:** 10.1186/1743-8977-9-16

**Published:** 2012-05-23

**Authors:** Linda Swedin, Romanico Arrighi, Britta Andersson-Willman, Ashley Murray, Yunying Chen, Mikael C I Karlsson, Susanna Kumlien Georén, Alexey V Tkach, Anna A Shvedova, Bengt Fadeel, Antonio Barragan, Annika Scheynius

**Affiliations:** 1 Institute of Environmental Medicine, Division of Molecular Toxicology, Karolinska Institutet, Stockholm, Sweden; 2 Swedish Institute for Communicable Disease Control, Stockholm, Sweden; 3 Center for Infectious Medicine, Department of Medicine, Karolinska Institutet, Stockholm, Sweden; 4 Translational Immunology Unit, Department of Medicine Solna, Karolinska Institutet, Stockholm, Sweden; 5 Pathology and Physiology Research Branch, Health Effects Laboratory Division, National Institute for Occupational Safety and Health, Center for Disease Control and Prevention, Morgantown, WV, USA; 6 Department of Clinical Science, Intervention and Technology, Division of ENTdiseases, Karolinska Institutet, Stockholm, Sweden; 7 Department Physiology and Pharmacology, School of Medicine, West Virginia University, Morgantown, WV, USA

**Keywords:** Carbon nanotubes, Bioluminescence imaging, Inflammation markers, Lung and spleen immunohistology, Dendritic cells, Macrophages, *Toxoplasma gondii*

## Abstract

**Background:**

Single-walled carbon nanotubes (SWCNT) trigger pronounced inflammation and fibrosis in the lungs of mice following administration via pharyngeal aspiration or inhalation. Human exposure to SWCNT in an occupational setting may occur in conjunction with infections and this could yield enhanced or suppressed responses to the offending agent. Here, we studied whether the sequential exposure to SWCNT via pharyngeal aspiration and infection of mice with the ubiquitous intracellular parasite *Toxoplasma gondii* would impact on the immune response of the host against the parasite.

**Methods:**

C57BL/6 mice were pre-exposed by pharyngeal administration of SWCNT (80 + 80 μg/mouse) for two consecutive days followed by intravenous injection with either 1x10^3^ or 1x10^4^ green fluorescence protein and luciferase-expressing *T. gondii* tachyzoites. The dissemination of *T. gondii* was monitored by *in vivo* bioluminescence imaging in real time for 7 days and by plaque formation*.* The inflammatory response was analysed in bronchoalveolar lavage (BAL) fluid, and by assessment of morphological changes and immune responses in lung and spleen.

**Results:**

There were no differences in parasite distribution between mice only inoculated with *T. gondii* or those mice pre-exposed for 2 days to SWCNT before parasite inoculum. Lung and spleen histology and inflammation markers in BAL fluid reflected the effects of SWCNT exposure and *T. gondii* injection, respectively. We also noted that CD11c positive dendritic cells but not F4/80 positive macrophages retained SWCNT in the lungs 9 days after pharyngeal aspiration. However, co-localization of *T. gondii* with CD11c or F4/80 positive cells could not be observed in lungs or spleen. Pre-exposure to SWCNT did not affect the splenocyte response to *T. gondii.*

**Conclusions:**

Taken together, our data indicate that pre-exposure to SWCNT does not enhance or suppress the early immune response to *T. gondii* in mice.

## Background

Carbon nanotubes (CNT), with their unique physico-chemical properties, yield numerous technological advantages for novel applications that are expected to drive industrial growth. As a consequence, large quantities of CNTs may reach the environment, and this may inadvertently result in human exposure. Hence, there is an urgent need for the assessment of potential impacts and health effects of CNTs [1]. The unusual properties of CNTs may underlie unique biological activities that may be exploited for biomedical applications, but insufficient research has been undertaken to explore their potential adverse effects on human health. Our earlier studies have shown that SWCNT induced a robust acute inflammation with a very early onset of the formation of granulomas and interstitial fibrosis in the lungs of C57BL/6 mice [2,3]. Several lines of evidence suggested that exposure to respirable CNT could modulate innate immunity and intervene with host resistance to microbial infections [2-5]. Thus, we have previously shown that exposure to SWCNT followed by *Listeria monocytogenes* (LM) infection resulted in decreased pulmonary clearance of bacteria [2].

*Toxoplasma gondii* is an obligate intracellular protozoan parasite that infects virtually all warm-blooded vertebrates. Up to one third of the global human population is chronically infected [6]. Following primary infection, the tachyzoite stage of the parasite disseminates widely in the organism. Differentiation of tachyzoites into tissue cyst stages (bradyzoites), predominantly in the brain, results in chronic asymptomatic infection. *T. gondii* is an opportunistic pathogen and reactivation of the infection can be lethal in individuals with acquired immune deficiencies, e.g. HIV/AIDS, or in individuals with prolonged treatments with immune suppressive drugs, e.g. recipients of organ and bone marrow transplants. Severe manifestations include toxoplasmic encephalitis [6-8] and neurological damage in the developing fetus [6]. Infection in the airways can manifest as severe atypical pneumonia [9-11].

The onset of cell-mediated immunity against *T. gondii* is accompanied by the transformation of the parasite into tissue cysts resulting in lifelong chronic infection. Cellular immunity mediated by NK cells, T cells, dendritic cells (DC), macrophages, and activity of type 1 cytokines (IL-12 and interferon (IFN)-γ) are essential to resist primary infection and for maintenance of quiescence during latent infection [12,13]. Mounting evidence indicates that the inherent migratory functions of leukocytes also make them a suitable target (Trojan horse) for *T. gondii* to mediate its dispersion in the organism [14-16].

Rodents are natural hosts for *T. gondii* and offer a robust infection model [15]. The aim of the present study was to determine whether pre-exposure of mice to SWCNT using the same pharyngeal aspiration protocol as in the infection model with the bacteria *L. monocytogenes*[2] would affect the dissemination and host response towards the parasite *T. gondii*. To evaluate the pathobiology of the Toxoplasma infection *in vivo,* we used bioluminescence imaging (BLI) which provides a versatile tool for non-invasive assessment with fine temporal resolution [17,18]. We also aimed to investigate whether pre-exposure of mice to SWCNT and *T. gondii* infection would alter the inflammatory responses, measured as cell counts and the cytokine profile in bronchoalveolar lavage (BAL) fluid, and in lung and spleen histology changes. The data presented here suggest that inhalation of SWCNT before encountering Toxoplasma infection do not interfere with the early immune response to *T. gondii*.

## Results

### Pre-administration of SWCNT does not affect the parasitic loads in the spleen and in the lung

**Figure 1:**
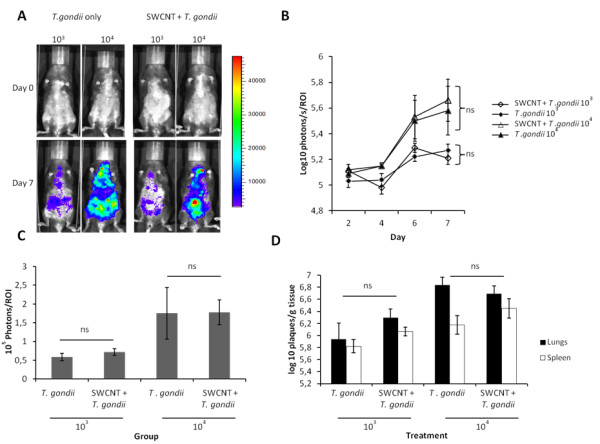
**Effects of pre-adminstration of SWCNT on the replication and dissemination of *T. gondii. *A**. Photonic emissions were assessed by bioluminescence imaging (BLI) on day 0 and 7 after parasite inoculation of mice with two different doses (10^3^ and 10^4^). The colour scale indicates photon emission (photons/s/cm^2^/sr) from *T. gondii* biomass over a 300 s exposure. Data are representative of mice from day 0 and 7 post-infection from two independent experiments. **B**. Total photon emission analysis from individual mice from day 2–7 post-inoculation, shows a strong increase in parasite burden after day 4, but non-siginificant differences between *T. gondii* only or SWCNT + *T. gondii* (ns, One-way ANOVA, p > 0.05). Results are shown as mean ± SEM (n = 6). **C**. Photon emission analysis from individual mice lung region of interest (ROI) on day 7 post-inoculation shows non-significant differences between *T. gondii* only or SWCNT + *T. gondii* groups (ns, One-way ANOVA, p > 0.05). Results are shown as mean ± SEM (n = 6). **D**. Parasite load in lungs and spleen day 7 after *T. gondii* inoculation quantified by plaquing assays as indicated under Materials and Methods. Non-significant differences were observed between *T. gondii* only or SWCNT + *T. gondii* (ns, one-way ANOVA, p > 0.05). Results are shown as mean ± SEM (n=6).

In order to monitor the dissemination of the parasite by non-invasive real-time imaging, we utilized PTG Type II GFPluc tachyzoites. Mice were monitored over 7 days post *T. gondii* inoculation, and regardless of initial parasite inoculum, the presence of SWCNT did not significantly affect the parasites development or dissemination as measured by BLI (Figure 1A & B). The higher inoculation dose of *T. gondii* led to higher parasitic loads over time as expected. Furthermore, strong parasite signals could be clearly detected in the lungs for all parasite treated groups, with the strongest signals detected from the *T. gondii* 10^4^ and SWCNT + *T. gondii* 10^4^ groups (Figure 1A). Analysing total photonic emissions from each group of mice showed that although emissions increased over time, particularly after day 4, there was no clear difference in total parasite photonic emissions between *T. gondii* only and SWCNT + *T. gondii* treated mice (Figure 1B, One-way ANOVA, p > 0.05). The lungs were studied in more detail to determine whether there was a difference in photonic emissions in that region, but again, no clear differences were detected between *T. gondii* only and SWCNT + *T. gondii* groups on day 7 post-infection (Figure 1C, One-way ANOVA, p > 0.05). In fact, similar photonic counts between both groups were observed in mice from day 4 onwards (data not shown). Quantification of parasitic load by plaquing assays from lungs and spleens extracted day 7 post-infection confirmed that both organs were heavily infected, with the lungs showing a higher burden per gram tissue than the spleen. However, there was no significant difference in the burdens in relation to the mice pre-treated with SWCNT (Figure 1D, One-way ANOVA, p > 0.05). We conclude that the replication and dissemination of *T. gondii* in our mouse model is not obviously hindered or exacerbated by the pre-exposure to SWCNT.

### No significant alterations in inflammatory markers in bronchoalveolar lavage fluid following combination exposure

**Figure 2:**
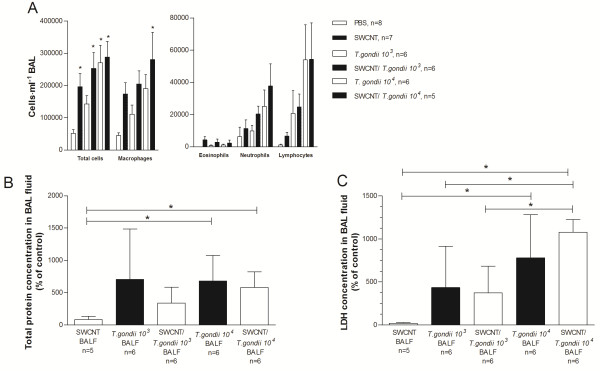
**Analysis of cell number and composition in BAL fluid. A **. Total number of cells and composition of cells in BAL fluid in C57BL/6 mice after exposure of SWCNT and *T. gondii * infection (n = 5-8). Data are presented as mean ± SEM. **B**. Protein content of BAL fluid. Results are presented as percentage of control (PBS treated mice) ± SD from triplicate of six mice divided into two experiments. **C**. LDH content of BAL fluid. Results are presented as percentage of control (PBS treated mice) ± SD from triplicate of five-six mice divided into two experiments. Statistical significance was determined with one-way ANOVA or Kruskal-Wallis test using Gaussian approximation and Bonferroni’s Multiple Comparison test. * = p < 0.05, indicate significant differences between groups. In panel A, * = p < 0.05 significant differences to PBS controls.

The combined effect of SWCNT treatment and *T. gondii* infection on the cellular inflammatory response was measured via the accumulation of leukocytes in BAL fluid. Following SWCNT challenge and *T. gondii* infusion, the total cell number and the number of most leukocytes subtypes, were markedly increased in BAL fluid (One-way ANOVA, p < 0.05; Figure 2A) compared to PBS controls. This was true for neutrophils, lymphocytes and especially for macrophages. When comparing the combined treatment with respective controls, i.e.*T. gondii* or SWCNT alone, there was a trend, albeit not significant, towards increased total cells and also the number of most leukocytes subtypes, with the exception of eosinophils (One-way ANOVA, p > 0.05; Figure 2A).

To determine cellular damage, total protein concentration and LDH was measured in BAL fluid. Exposure to only SWCNT induced minimal changes in the levels of total protein content and LDH levels compared to the PBS controls (Figure 2B and C). There was a significant increase in the total protein content and LDH levels in mice infected with the higher concentration of *T. gondii* (One-way ANOVA, p < 0.05)*.* However, there were no differences between the groups only exposed to *T. gondii* and those also pre-exposed to SWCNT, but a tendency to higher LDH levels could be observed for the combination in mice exposed to the higher concentration of *T. gondii* (Figure 2C).

To clarify the possible contribution of inflammatory mediator cascades in the airway inflammation, a 7-plex panel of cytokines, chemokines and growth factors and a 3-plex panel of TGFβ, were measured in BAL fluid. Exposure to only SWCNT generally induced no change in the levels of mediators in BAL fluid compared to PBS controls at day 10, except for IL-12p40 and TGFβ2, although not statistically significant (Table 1, one-way ANOVA and further analysed using Bonferroni’s Multiple Comparison post hoc test). Although there were increases in all mediators measured after *T. gondii* infection, these changes did not always reach statistically significant differences compared to PBS controls and mice exposed to SWCNT only, probably due to the small size of the groups (Table 1). Furthermore, there was no clear effect of the combined treatments of SWCNT and infection of *T. gondii.* The TGFβ-plex assay revealed increased levels of TGFβ1 in mice exposed to SWCNT and/or *T. gondii* compared to PBS controls, although not statistically significant (data not shown, one-way ANOVA). There were significantly increased levels of TGFβ2 in BAL fluid in mice exposed to SWCNT + *T. gondii* 10^4^ compared to PBS controls or mice exposed to SWCNT alone and compared to *T. gondii* 10^3^ alone or combined with SWCNT (Table 1, one-way ANOVA, p < 0.05). TGFβ3 levels were at the detection range for this particular mediator (data not shown).

**Table 1:** **Levels of cytokines, chemokines and growth factors in BAL fluid in mice exposed to SWCNT and inoculation with the parasite *T. gondii ***

**Group of animals (n = 6-8)**	**Cytokine release in BAL fluid (pg/mL)**							
	**IL-1β**	**IL-6**	**IL-10**	**IL-12p40**	**INFγ**	**TNFα**	**MCP-1**	**TGFβ2**
PBS	55 ± 29	18 ± 9	34 ± 12	458 ± 67	45 ± 9	48 ± 30	61 ± 37	84 ± 10
SWCNT	8 ± 0	18 ± 7	10 ± 4	1173 ± 146	25 ± 9	9 ± 2	51 ± 22	165 ± 23
*T. gondii* 10^3^	432 ± 227*	408 ± 136	552 ± 236^#^*	3468 ± 688^#^*	5769 ± 1799^#^*	1016 ± 466	2569 ± 978	124 ± 17
SWCNT/ *T. gondii* 10^3^	165 ± 56	497 ± 84	502 ± 108	3752 ± 292^#^*	9090 ± 1807^#^*	720 ± 199	3558 ± 745	187 ± 22
*T. gondii* 10^4^	229 ± 40	1058 ± 208^#^*	612 ± 130^#^*	2587 ± 409^#^	8977 ± 966^#^*	859 ± 246	8214 ± 2180^#^*	286 ± 58^#$^
SWCNT/ *T. gondii* 10^4^	288 ± 51	1186 ± 387^#^*	798 ± 138^#^*	2696 ± 565^#^	7245 ± 1023^#^*	1048 ± 296*	11406 ± 2167^#^*^$*f*^	358 ± 52 ^#^*^$*f*^

Results are presented as mean ± SEM. Statistical significance was determined with one-way ANOVA and Bonferroni’s Multiple Comparison test. # = p < 0.05 indicates significant differences from PBS, * = p < 0.05 indicates significant differences from SWCNT, $ = p < 0.05 indicates significant differences from *T. gondii* 10^3^, and *f* = p < 0.05 indicates significant differences from SWCNT + *T. gondii* 10^3^. BAL fluid samples were obtained at 7 days post-exposure.

### Co-exposure to *T. gondii* and SWCNT yields no novel histological changes in the lungs as compared to mice infected with *T. gondii* or exposed to SWCNT alone

**Figure 3:**
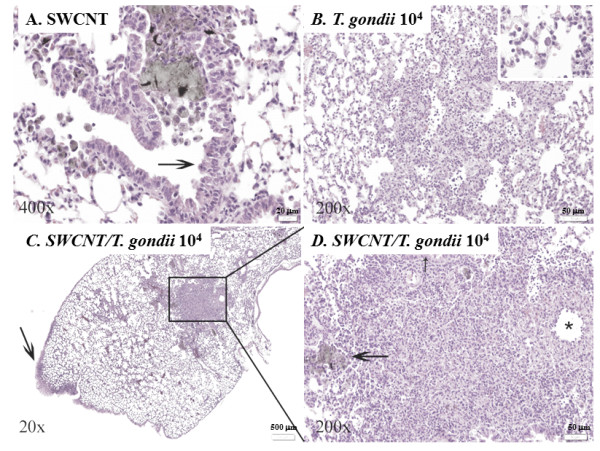
**Pulmonary inflammation after SWCNT and/or*T. gondii*exposure.** Representative photomicrographs of pulmonary changes in mouse lungs. **A**. High magnification image of an airway containing a large amount of pigmented particulate matter both free and within macrophages. The macrophages surround the material forming a small granuloma and spread out of the airway into adjacent alveoli. There is mild epithelial hyperplasia (arrow). **B**. High magnification view of a lung characteristic of those infected with *T. gondii* with loss of alveolar spaces due to pyogranulomatous inflammation and alveolar necrosis with intra-lesional protozoal vacuoles (inset). **C**. Lung from a mouse administered SWCNT and infected with the higher dose of *T. gondii.* There is focally extensive pleuritis (arrow), one large area of inflammation obliterating normal pulmonary architecture (box) and multifocal smaller nodules. **D**. Higher magnification view of the boxed area in (**C**) shows a combination of particle-laden macrophages filling airways (large arrow) and mild epithelial hyperplasia (small arrow). This is combined with vasculitis (asterisk) and necrosis with a pyogranulomatous inflammatory cell infiltrate as seen in the *T. gondii* infected animals.

**Figure 4:**
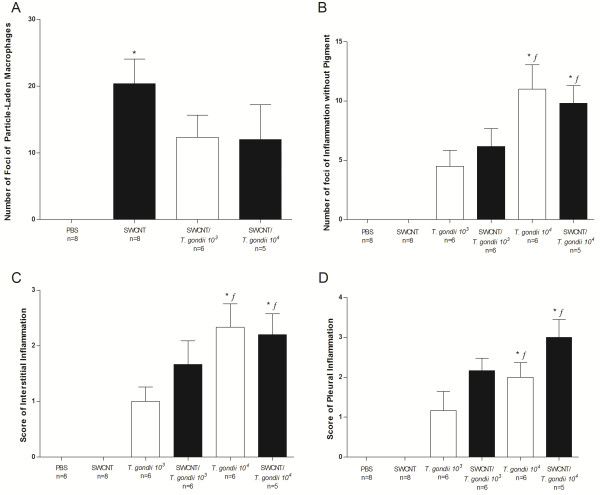
**Histological findings in lung tissue of exposed animals. A **. The number of foci of particle-laden macrophages forming granulomas in the lungs. Animals infected with *T. gondii * alone were not included as no foci of particle-laden macrophages were observed. **B **. The number of foci of necrosis and inflammation unassociated with pigment and consistent with *T. gondii * infection. **C**. Difference in interstitial inflammation scores for the various groups. **D **. Pleuritis scores for each group. Data are represented as mean ± SEM. Statistical significance was determined with Kruskal-Wallis one-way ANOVA and Dunn’s post-test. * = p < 0.05, indicate significant differences from PBS controls and *f * = p < 0.05, indicate significant differences from SWCNT.

To further examine the effect of a combined treatment of SWCNT and infection of *T. gondii* on the inflammatory response, histopathology of the lungs was performed on hematoxylin/eosin stained sections from formalin fixed specimens. The PBS control mice were unaffected and all had normal lungs. The lungs of mice exposed to only SWCNT contained variable numbers of foci of particle-laden macrophages forming distinct granulomas (Figure 3A). SWCNT-treated animals had between 0 and 33 (mean = 20.4) foci of particle-laden macrophages in their lungs (Figure 4A). These foci generally originated in bronchi and bronchioles and extended into alveoli from these airways (Figure 3A). The two groups of mice infected with *T. gondii* alone showed no evidence of granulomas as seen in the SWCNT group, but rather had multiple foci of necrotizing pyogranulomatous pneumonia (Figure 3B). Inflammation extended into the alveolar interstitium and multifocally involved the pleural surface with variable degrees of pleuritis (Figure 3B and 4B-D). Animals given higher doses of *T. gondii* organisms were more severely affected (Figure 4B-D). In animals administered both SWCNT and *T. gondii* there was a mixture of those lesions seen in the SWCNT-treated and *T. gondii*-infected animals with foci of particle-associated granulomas and foci of necrotizing pneumonia (Figure 3C and D and Figure 4). For animals co-infected with the lower dose of *T. gondii*, animals had scores between 0 and 22 (mean = 12.3). At a higher dose of *T. gondii*, animals had between 0 and 30 (mean = 12) foci of particle-laden macrophages in their lungs (Figure 4A). Both SWCNT + *T. gondii* groups approached significance and a higher number of animals would likely have confirmed statistically the obvious decrease in pigmented foci. In some areas, these lesions were distinct from one another while in others they overlapped. The animals also displayed pleuritis due to the *T. gondii* infection (Figure 4D). In general, the number of necroinflammatory foci was dependent on the infectious dose of *T. gondii* and did not depend on the administration of SWCNT. While animals administered *T. gondii* and SWCNT had fewer particle-associated granulomas overall, this could partially be explained by larger sized lesions with fewer distinct granulomas as shown by the percent alveolar loss being markedly higher in the jointly treated groups than those administered SWCNT alone (data not shown).

**Figure 5:**
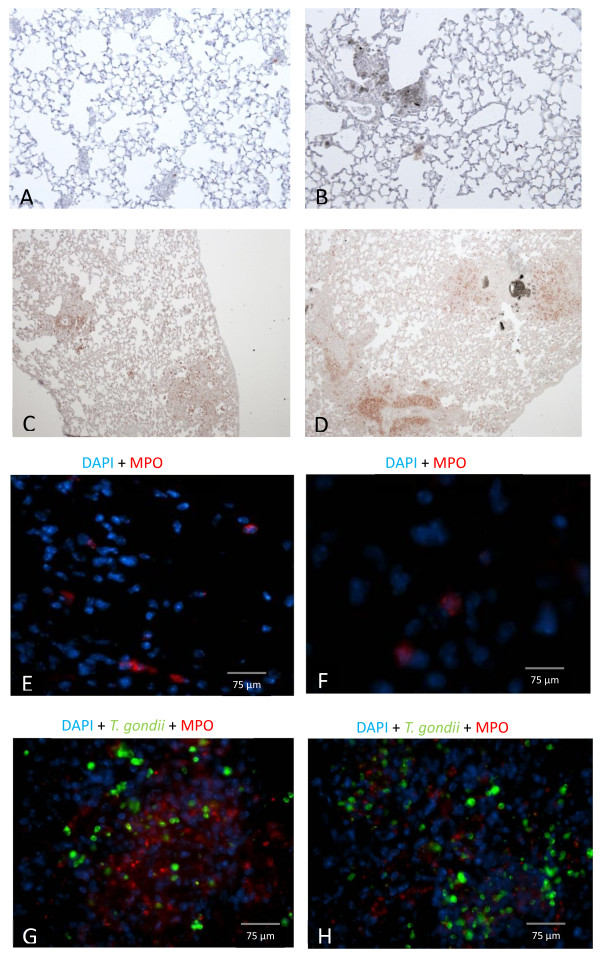
**Co-localisation of MPO positive neutrophils and *T. gondii *in lung tissue. A-D **. Histological staining of MPO positive neutrophils in lung tissue from C57BL/6 mice after exposure of SWCNT and/or *T. gondii * inoculation. Representative pictures of (**A**) PBS control, 40x magnification (**B**) SWCNT, 40x magnification (**C **) *T. gondii * 10^4^, 20x magnification (**D**) SWCNT + *T. gondii* 10^4^ 20x magnification. **E-H**. Representative photomicrographs of fluorescence stained cells within pulmonary tissue of (**E**) PBS control, (**F**) SWCNT, (**G **) *T. gondii * 10^4^, (**H **) SWCNT + *T. gondii * 10^4^. Sections were mounted in medium for fluorescence including DAPI (blue). MPO positive neutrophils stained in red and *T. gondii* parasites stained in green in lung tissue.

When examining MPO positive neutrophils, it was clear that differences occurred between mice that were exposed to SWCNT and mice infected with *T. gondii* (Figure 5A-D). Whereas there was an extensive accumulation of neutrophils in the granulomas from mice infected with *T. gondii*, only a few neutrophils were detected in the granulomas from mice exposed only to SWCNTs. Lung sections from mice infected with *T. gondii* clearly showed that MPO positive neutrophils were concentrated to the more extensive granulomas, most likely as a result from *T. gondii* infection. When examining co-localization of MPO positive neutrophils and *T. gondii,* it was revealed that MPO positive neutrophils could be detected in neither the PBS control group nor the SWCNT exposure group (Figure 5E-H). Even though there were many more MPO positive neutrophils in the presence of *T. gondii,* there were no obvious differences between SWCNT pre-treated mice infected with *T. gondii.* The staining of MPO positive neutrophils was more intense with the higher parasite dose and the MPO staining was quite intense in the vicinity of high parasite burdens (Figure 5E-H). Parasites were present in both *T. gondii* groups (presence/absence of SWCNT), with no difference between the two *T. gondii* concentrations (Figure 5G-H). The presence of large intracellular vacuoles following lung section staining with anti-toxoplasma Ab is indicative of parasite replication (data not shown).

**Figure 6:**
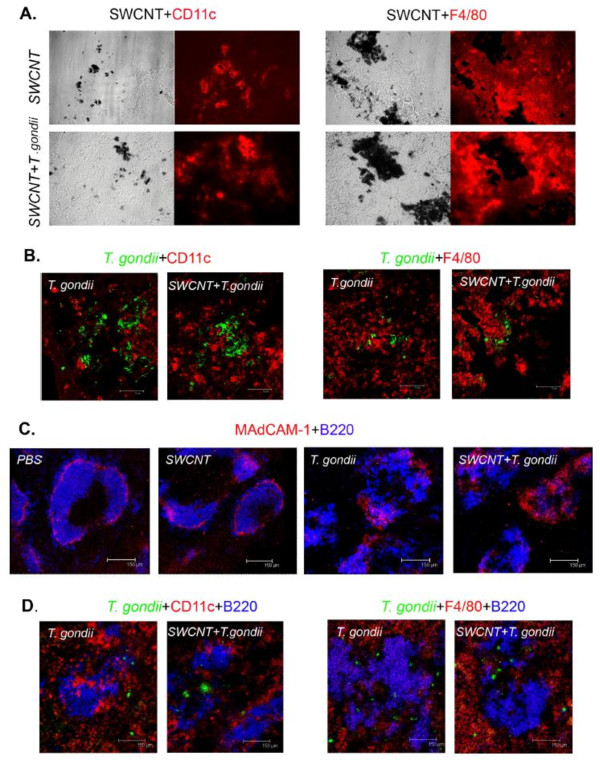
**Co-localization of dendritic cells and/or macrophages with SWCNT or *T. gondii *in spleen and lung.** Representative pictures of **A **. visualization of nanoparticles and CD11c or F4/80 positive cells in lungs of both SWCNT and SWCNT + *T. gondii * 10^4^ treated mice. 40x magnification **B **. Double staining with anti-toxoplasma Ab and anti-CD11c or anti-F4/80 mAb in lungs of both SWCNT and SWCNT + *T. gondii * 10^4^ treated mice. **C**. MAdCAM-1 and B-cell staining (B220) in frozen spleen sections. **D**. Triple staining with anti-toxoplasma, anti-B220 and anti-CD11c or anti-F4/80 in frozen spleen sections.

To study co-localization of dendritic cells and/or macrophages with SWCNT or *T. gondii*, double immunofluorescence staining of lung sections was performed. The SWCNT in lungs were visualized by light microscopy. The SWCNT co-localized with CD11c positive dendritic cells (DCs) in lungs of both SWCNT and SWCNT ± *T. gondii* treated mice, indicating that CD11c positive DCs can engulf the SWCNT (Figure 6A); however, the particles did not co-localize with F4/80 positive cells (macrophages). The F4/80 positive macrophages accumulated around the SWCNT and participated in the formation of granuloma. Double staining with anti-toxoplasma Ab and anti-CD11c or anti-F4/80 mAb showed that the CD11c and F4/80 positive cells did not co-localize with *T. gondii* in lungs from mice irrespective of SWCNT pre-treatment (Figure 6B).

To further study lung pathology, PAS staining was performed to assess mucin-containing goblet cells. There was a slight increase in the number of goblet cells in mice challenged with SWCNTs based on representative lung sections whereas hardly any goblet cells were detected in control mice or mice infected with *T. gondii* (data not shown).

### Pre-exposure to SWCNT did not affect the splenocyte response to *T. gondii* localized in both the follicle and red pulp of spleen

To investigate systemic effects of SWCNT on *T. gondii* infection, spleen tissue were examined. MAdCAM-1 staining was used to show the marginal sinus structure of spleen, which separates the follicle (inside) and red-pulp (outside). Double immunofluorescence staining showed that toxoplasma infection impaired the structure of the spleen. The marginal sinus and follicles in *T. gondii* infected mice showed a defective structure compared with mice receiving PBS or SWCNT (Figure 6C). Triple staining with anti-toxoplasma, anti-B220 (B cell marker) and anti-CD11c or anti-F4/80 showed that *T. gondii* localized in both the follicle and red pulp of spleen; no co-localization (double staining) was seen with anti-toxoplasma and anti-CD11c or anti-F4/80 mAb (Figure 6D). Furthermore, there was no co-localization between the MARCO (scavenger receptor) positive macrophage population and *T. gondii* or SWCNT (data not shown).

**Figure 7:**
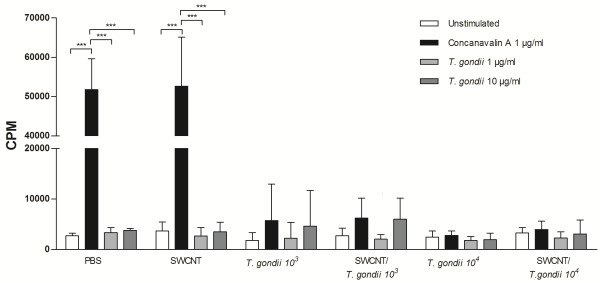
**Impaired immune response after *T. gondii *exposure. ** Single cell suspensions from spleens from mice exposed to SWCNT and/or *T. gondii * in two doses (10^3^ or 10^4^ parasites) were cultured for 66 h with or without ConA (1 μg/ml) or *T. gondii* antigen (1 or 10 μg/ml). ^3^[H]-Thymidine was added during the last 18 h and incorporation was determined by scintillation counting. Results are expressed as mean counts per minute (cpm) from six mice/group ± SD. Level of significance *** p < 0.001 determined with two-way ANOVA, and Bonferroni’s Multiple Comparison test.

Splenocytes from PBS control mice and mice exposed to only SWCNT showed an increased proliferation to ConA, but did not respond to the *T. gondii* antigen as expected (Figure 7). However, spleen cells from mice exposed to *T. gondii* with or without pre-exposure to SWCNT demonstrated a decrease in cell proliferation after ConA and *T. gondii* antigen stimulation (Figure 7). The quality of the *T. gondii* antigen was confirmed with the release of IFN-γ into the cell culture medium of single cell suspensions from spleens of *T. gondii* infected mice (See Additional file 1; [19]).

## Discussion

It has been previously reported that pulmonary exposure to SWCNT induced robust inflammation, early granulomatous lesions and interstitial fibrosis in the lungs of mice [2,3]. The histological data obtained from the current study exposing mice to respirable SWCNT are very consistent with the previously reported (Figure 4). Notably, the architecture of mouse lungs after exposures to SWCNT and SWCNT + *T. gondii* groups demonstrated an increase in pigmented foci after exposure to SWCNT and SWCNT + *T. gondii*. No granulomatous lesions were observed in lungs of mice treated with *T. gondii*. There were also no differences in severity of granulomatous lesions seen in SWCNT and SWCNT + *T. gondii* exposure groups. Several recent reports indicated that SWCNT exposure was able to modify outcomes to infectious agents. In particular, it was demonstrated that sequential exposure to SWCNT and *L. monocytogenes* (LM) amplified lung inflammation and collagen deposition in mouse lungs [2]. The pulmonary clearance of LM from the lungs of SWCNT pre-exposed animals was markedly delayed. The reduced ability of macrophages to phagocytose LM in the presence of SWCNT was found to be related to a reduction of macrophage production of nitric oxide in response to LM [2]. However, it has to be emphasized that in the LM + SWCNT study, both agents were concomitantly administered to mice via aspiration route. While *T. gondii* can elicit pathology in the pulmonary tissues [9-11], inhalation is not the natural route of access. During natural infections or reactivated acute infection, the parasite reaches the lung tissue through the systemic circulation. In contrast with the administration route from the LM study, we infected our mice intravenously with *T. gondii*, providing widespread haematogenic dissemination of parasites and enhanced parasite infection in the lungs compared to other administration routes [17]. Even with enhanced parasite migration, the lungs allowed for a better assessment of parasite replication as opposed to other organs such as the spleen, where replication is usually much more extensive. Thus, despite haematogenous access of parasites to lung tissue, the parasitic loads in SWCNT-exposed mice were not significantly higher compared to untreated mice. To this end, our current data have revealed that the progression of an initially generalized *T. gondii* infection was not significantly modified by an acute pulmonary response to respirable SWCNT administrated prior to the infection. Reciprocal approaches have shown that immune responses to *T. gondii* can mediate protective effects on concomitant inflammatory conditions in the lungs [20-22] and are indicative of the complexity of the immune response. In contrast, the experimental protocol applied here assessed whether the host response following pharyngeal aspiration of SWCNT affects *T. gondii* infection. In our study, pre-exposure to SWCNT did not enhance or suppress the early immune response to *T. gondii* in mice. Rather, the absence of accentuated pathology in the lungs indicates that the deposition of SWCNT in the lung tissue may have a different impact on immune responses depending on the route of access for the pathogens, with a relatively minor impact on haematogenously spread infection compared to airway exposure.

The selected dose range for exposure to respirable SWCNT (160 μg/mouse) in the current study was based on previously found immune suppression reported in mice [20]. The calculated exposure levels in this study are relevant to those found in actual workplace conditions and, are in fact lower than those levels that could be achieved during life-time work exposures [23]. The cumulative SWCNT doses we used were given to mice on day 1 and 2 and *T. gondii* was administered on day 3. However, further investigations are warranted to determine whether prolonged pre-exposure to SWCNT would have a more discernible impact on the establishment and development of *T. gondii* infection *in vivo*.

Several lines of evidence suggest that migrating DC play a critical role during *T. gondii* infection as systemic carriers of *T. gondii* tachyzoites [14,16,24,25]. In addition, DCs function as both antigen-presenting cells as well as the main source of IL-12 and TNF-α in response to *T. gondii* antigens [26]. IL-12 induces early IFN-γ production in NK cells and facilitates subsequent Th1 development, whereas TNF-α is involved in mediating resistance to acute and chronic *T. gondii* infections [27-30]. Infection with *T. gondii* did not give any immediate sign of unhealthyness but there was a significant weight loss by day 10 in the *T. gondii* infected mice without any differences related to whether mice had been pre-exposed to SWCNT or not (See Additional file 2). Our measurements of inflammatory mediators in BAL fluid showed that there were some increases in several mediators measured after *T. gondii* infection, albeit not statistically significant when compared to PBS controls and mice exposed to SWCNT only. Furthermore, there was no clear difference following co-exposure to SWCNT and *T. gondii.* Exposure to SWCNT alone induced no changes in the levels of mediators in BAL fluid. Hence, while it has been clearly established that infection with *T. gondii* results in interplay between the parasite and the host [31], resulting in a complex cascade of pro- and anti-inflammatory responses [32], we could not reveal any effects of SWCNT exposure on the parasite-induced responses.

Notably, we have recently found that SWCNT-induced inflammation facilitated the recruitment of DCs to the lung and redistribution of DCs to lymphoid tissues [23]. In the present study, nanoparticles co-localized with CD11c positive DCs in lungs of both SWCNT and SWCNT ± *T. gondii* treated mice, however, they did not co-localize with F4/80 positive cells (macrophages), indicating that CD11c positive DC interact with the particles. We have previously shown uptake of SWCNT by DCs *in vitro* using murine DCs generated from hematopoietic progenitors isolated from bone marrow [23]. Our finding here on the *in vivo* uptake of SWCNT in the lung by CD11c positive DCs but not by F40/80 positive macrophages is to our knowledge novel data. Moreover, previous findings [28,33] indicate that infected DCs are compromised in their ability to activate T cells, suggesting that the arrival of infected DCs in lymphoid tissues will not result in efficient priming of a T cell response. In our recent study, direct effects of SWCNT on DCs resulted in suppressed spleen T cell responses upon pulmonary exposure [23]. Pulmonary exposure to multi-walled carbon nanotubes (MWCNT) was also reported to suppress spleen cells responses to mitogen stimulation (17, 18). In the current study, we observed even greater suppression of splenocyte proliferation in all groups infected with *T. gondii*, which has been described previously [19]. In addition, it was found that *T. gondii* was not taken up by the macrophage (F4/80 or MARCO positive) or DCs (CD11c positive) populations in the spleen. Unfortunately, the data for SWCNT was not conclusive since the spleen is filtering red blood cells resulting in iron deposits thus making it impossible to specifically detect the nanoparticles using light microscope. It remains to be investigated which population of cells respond to *T. gondii* infection and produce IFNγ in the spleen. However, there was no evidence of any modulatory effect on *T. gondii* proliferation or dissemination in the host in the presence of SWCNT in the current model.

As expected, the lungs of mice exposed to only SWCNT contained variable numbers of foci of particle-laden macrophages forming distinct granulomas. The two groups of mice infected with *T. gondii* alone showed no evidence of granulomas; instead, multiple foci of necrotizing pyogranulomatous pneumonia were found in the lung, and mice receiving higher doses of *T. gondii* organisms were more severely affected. The co-exposure to *T. gondii* and SWCNT appeared to yield a mixture of histological changes in the lung with foci of particle-associated granulomas as well as foci of necrotizing pneumonia. In general, however, the number of necroinflammatory foci was dependent on the infectious dose of *T. gondii* and did not depend on the administration of SWCNT. In addition, the increased numbers of neutrophils were a result of inoculation of *T. gondii* rather than SWCNT exposure, whereas CD11c positive DC co-localized with SWCNT exposure possibly due to engulfment of the particles.

## Conclusions

In synopsis, the current analysis of infection parasite distribution and parasite burden using non-invasive BLI as well as plaquing assays shows that administration of SWCNT via pharyngeal aspiration prior to infection with *T. gondii* has no discernable effect on the establishment, dissemination, and proliferation of infection. This, however, does not preclude the possibility that exposure to SWCNT may affect the establishment of the chronic phase of infection. Further studies are warranted to address this question. In conclusion, our data suggest that exposure to SCWNT does not affect the early immune response against *T. gondii*.

## Materials and methods

### Animals and parasites

Female C57BL/6 mice (6–8 weeks of age) were purchased from Charles River (Sulzfeld, Germany) and maintained under pathogen-free conditions at the Swedish Institute for Communicable Disease Control animal facility (The animals were housed in ventilated filtered plastic cages with absorbent bedding material and were maintained on a 12 h daylight cycle). Food and water were provided *ad libitum*. All animal experiments were approved by the regional committee of animal experimentation ethics (Dnr: N15/11; Stockholm North ethical committee for animal welfare, Stockholm, Sweden).

Tachyzoites from the green fluorescence protein (GFP) and luciferase-expressing *T. gondii* type II line PTGluc (cloned from ME49/PTG-GFPS65T) [17] were maintained by serial 2-days passage in human foreskin fibroblast (HFF) monolayers. HFFs were propagated in Dulbecco's modified Eagle's medium (DMEM; Invitrogen, Carlsbad, CA, USA) with 10% fetal bovine serum (FBS), gentamicin (20 μg/ml, Gibco), glutamine (2 mM, Gibco) and Hepes (0.01 M, Gibco).

### Single walled carbon nanotubes

**Figure 8:**
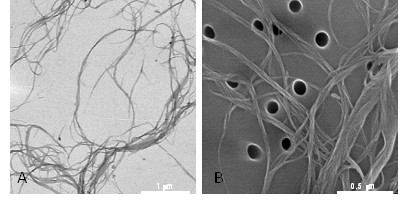
**Particle characterization. A**. Transmission electron microscopy (TEM) image of individual SWCNT confirmed a length of approximately 1–3 μm; **B**. Scanning electron microscopy (SEM) of the samples showed that sonication resulted in the production of well-dispersed SWCNT.

SWCNT (CNI, Houston, TX) produced by the high-pressure CO disproportionation (HiPco) process, employing CO in a continuous-flow gas phase as the carbon feedstock and Fe(CO)_5_ as the iron-containing catalyst precursor and purified by acid treatment to remove metal contaminants were used in the study. Morphology of the SWCNT is presented in Figure 8 and the detailed characterization of the utilized SWCNT is provided elsewhere [23]. Stock suspensions (1 mg/ml) were prepared before each experiment in PBS or culture medium and pH was adjusted to 7.0. Endotoxin content in SWCNT samples was assessed using the Limulus amebocyte lysate (LAL) enzyme assay (DataChem Inc, Salt Lake City, UT). The endotoxin content of SWCNT suspensions was lower than 0.11 EU/ml. The amount of endotoxin received by mice was 0.006 EU, a dose equivalent to 0.6 pg endotoxin. A similar level of endotoxin was found in the vehicle (pharmaceutical grade). To obtain a more homogenous and dispersed suspension, SWCNT were ultrasonicated (30 sec × 3 cycles). Scanning electron microscopy of the samples showed that sonication resulted in the production of well-dispersed SWCNT (Figure 8B).

### Experimental design

The study was divided into two separate experiments starting on two consecutive days. Mouse pharyngeal administration was used for instillation of the SWCNT particles. Briefly, after anesthetization with a mixture of ketamine and xylazine (62.5 and 2.5 mg/kg, respectively), the mouse was placed on a board in a near vertical position and the tongue was extended with forceps. A suspension of 40 μl particles in PBS was placed in the throat, and the tongue held until the suspension was aspirated into the lungs. A total dose of 160 μg/ mouse (80 + 80 μg) or PBS was aspirated on day 1 and 2. At day 3, the mice were infected intravenously in the tail with either 1 × 10^3^ or 1 × 10^4^ freshly egressed tachyzoites resuspended in 200 μl PBS (PTG-GFPluc). Control mice were injected intravenously in the tail with 200 μl PBS only. The six experimental groups (n = 6-8 per group) were: PBS-animals pre-treated and injected with PBS; SWCNT- animals pre-treated with 160 μg SWCNT and injected with PBS; *T. gondii* 10^3^-infected animals pre-treated with PBS; SWCNT + *T. gondii* 10^3^-infected animals pre-treated with 160 μg SWCNT; *T. gondii* 10^4^-infected animals pre-treated with PBS; and SWCNT + *T. gondii* 10^4^-infected animals pre-treated with 160 μg SWCNT. On day 10, the weight of the animals was assessed. Thereafter, the animals were sacrificed immediately whilst under anaesthesia via cervical dislocation, bronchoalveolar lavage (BAL) was performed, followed by dissection of the spleen and lungs.

### *In vivo* bioluminescence imaging (BLI)

BLI was performed 2, 4, 6 and 7 days after *T. gondii* injection, as described previously [17]. Briefly, mice were injected i.p. with 3 mg D-luciferin potassium salt (Caliper Life Sciences, Hopkinton, MA, USA) and anaesthetized with 2.3% isoflurane prior to BLI. Ten min after injection of D-luciferin, biophotonic images were acquired at a binning of 8 (medium) for 300 s with an In Vivo Imaging System Spectrum (IVIS Spectrum Caliper Life Sciences, Hopkinton, MA, USA). Analysis of images and assessment of photons emitted from a region of interest (ROI) was performed with Living Image software (version 3.2; Caliper Life Sciences, Hopkinton, MA, USA).

### Bronchoalveolar lavage (BAL)

BAL was performed after IVIS measurements on day 10. A total volume of 1 ml PBS was used to lavage the lungs via trachea, by using a prefilled 1 ml syringe which was inflated and aspirated three times with the same fluid. When required, red blood cells were removed by resuspending the BAL fluid cells in 100 μl lysis buffer (150 mM NH_4_Cl, 10 mM KHCO_3_, 0.1 mM EDTA, pH 7.2) for 2 min at RT followed by washing in 1 ml PBS. The total number of cells was then counted and adjusted to cells·ml^-1^ BAL fluid. For differential cell counts, cells were stained with May Grünwald-Giemsa and a minimum of 300 cells were counted per BAL fluid sample.

### Protein concentration

The total protein concentration in BAL fluid was measured with the BioRad DC protein assay (BioRad Life Science Research, Hercules, CA, USA) according to manufacturer’s instruction. Triplicate of samples were read at 650 nm using a Spectrophotometer (Multiskan Ascent, Thermo Scientific, Waltham, MA, USA). Results are presented as % of control treated mice ± SD.

### Lactate dehydrogenase (LDH)

To determine the release of LDH in BAL fluid, the CytoTox 96® Non-Radioactive Cytotoxicity Assay (Promega Corporation, Madison, WI, USA) was used. Triplicates of BAL fluid aliquots (50 μl, 2 times diluted in PBS) was transferred to a 96-well flat bottom plate. Assay buffer and substrate was mixed according to manufacturer’s instruction to constitute Substrate mix. 50 μl of Substrate mix was added to all wells and the plate was incubated in dark in a humidified atmosphere at 37°C. After 30 min incubation, 50 μl of stop solution was added to all wells and the absorbance was recorded at 492 nm using a Spectrophotometer (Multiskan Ascent, Thermo Scientific, Waltham, MA, USA). Results are presented as % of control treated mice ± SD.

### Plaquing assays

Directly after BAL was done on day 10, the lungs and spleens were extracted. Organ homogenization (half of the spleen and one lobe on the right side of the lung) was performed under conditions that did not affect parasite viability [34]. The number of viable parasites was determined by plaque formation on human foreskin fibroblast monolayers as described [34].

### Measurement of released mediators in BAL fluid

The levels of selected cytokines/chemokines and growth factors were measured in duplicates in BAL fluid using a mouse plex-assay (BioRad, Hercules, CA, USA) and assayed by Luminex® (BioRad, Hercules, CA, USA). Labelled antibodies against IL-1 β, IL-6, IL-10, IL-12p40, MCP-1, IFN-γ TNF-α and TGF-β1-3 were used for analyses of multiple cytokine responses according to provided instructions. Results below the detection limits were set as the absolute threshold for statistical evaluation.

### Lung histology

Left lung was inflated with 4% formalin (Sigma-Aldrich, St. Louis, MO, USA) at a pressure of 20 cmH_2_O and fixed for 24 h. Five μm-thick lung sections were cut and assessed with hematoxylin and eosin to evaluate the extent of cellular infiltration in the lung tissue. Slides (one section per lung, several fields of view for each slide to secure the conclusions) were examined under non-coded conditions and were assessed for histopathologic changes. Bronchiolar epithelial hyperplasia was scored on a scale from 0–5, where 0 = no change and 5 = severe change. The number of foci of particle-laden macrophages were counted in the samples. For animals infected with *T. gondii*, the number of necroinflammatory foci were counted, the degree of bronchiolar hyperplasia, pleuritis, and the amount of interstitial inflammation were scored on a scale of 0–5 as above. For animals infected with *T. gondii* and administered SWCNT, a combination of findings were recorded including counts of the number of foci of particle-laden macrophages, necroinflammatory foci and scores for bronchiolar hyperplasia, pleuritis, and interstitial inflammation. In addition, slides were stained with periodic acid-Shiffs staining (Sigma-Aldrich, St. Louis, MO, USA) to assess mucin-containing goblet cells.

### Immunohistochemical stainings of lung and spleen

Immunohistochemical staining for detection of *T. gondii* and neutrophils (myeloperoxidase, MPO) on the formalin fixed lung sections was performed. Initially, lung sections were hydrated with tap water, followed by antigen retrieval, pretreatment of 3% hydrogen peroxide and 2.5% normal horse serum (Vector Labs, Burlingame, CA, USA) to block nonspecific reactivity. Sections were incubated with rabbit-anti-mouse MPO (ab9535, AbCam, Cambridge, UK; 1:100, 4°C overnight) and with a peroxidase conjugated anti-rabbit as secondary antibody (ImmPRESS, MP-7401, Vector labs, Burlingame, CA, USA; 30 min, RT). After washing, color was developed by adding AEC (3-amino-9-ethylcarbazole) chromogen for 10 min (SK-4200, Vector Labs, Burlingame, CA, USA). Finally, slides were counterstained with hematoxylin and mounted using faramount (DAKO, Glostrup, Denmark). Negative control sections were treated in the same way but primary antibodies were omitted. Sections were also double-stained with rabbit-anti-mouse MPO (ab9535, AbCam, Cambridge, UK; 1:100, 4°C ON) primary antibody and primary human-polyclonal anti-*T*. *gondii* antibodies (1:500, WHO’s standard, Statens Serum Institute, Copenhagen, Denmark) followed by a secondary anti-rabbit Alexa 594 (1:400, Molecular Probes, Eugene, OR, USA) and Alexa Fluor 488 goat anti-human IgG (1:400, Molecular Probes, Eugene, OR, USA), respectively, to detect co-localization of MPO positive neutrophils and parasites. Sections were mounted in medium for fluorescence including DAPI (Vector labs, Burlingame, CA, USA). Stained sections were examined using light microscope and images were capture at 20x or higher magnifications.

In addition, 8 μm thin cryosections of snap frozen spleen or lung specimens were fixed in acetone. After blocking with goat serum (DAKO, Glostrup, Danmark) and avidin/biotin blocking kit (Vector labs, Burlingame, CA, USA), sections were incubated with primary Ab, followed by several washes in PBS and incubation with fluorescently-labelled secondary Abs or Alexa-555-conjugated streptavidin (Molecular Probes, Eugene, OR, USA). The following Abs were used: anti-mouse F4/80 (CI:A3-1, rat IgG2b, AbD Serotec, Oxford, UK); biotin conjugated anti-mouse.

CD11c (HL3, hamster IgG1, BD biosciences, San Jose, CA, USA), visualized by Alexa-555-streptavidin (Invitrogen, California, USA); anti-toxoplasma (WHO’s standard, Statens Serum Institute), anti-mouse MARCO (ED31, rat IgG1, AbD Serotec, Oxford, UK), Alexa-488-conjugated goat anti-human IgG (Molecular Probes, Eugene, OR, USA) Alexa-555-conjugated goat anti-rat IgG (Molecular Probes, Eugene, OR, USA). Stainings with isotype controls were performed to confirm the specificity of the immunostainings. Images were acquired using a confocal laser scanning microscope (TCS SP2; Leica Microsystems, Mannheim, Germany) or Leica application suite (Leica Microsystems).

### Splenocyte culture and proliferation

Spleen samples (one third of the spleen) were collected in complete medium consisting of RPMI 1640 medium (Sigma Aldrich, St. Louise, MO) supplemented with 2 mmol/L L-glutamine, 100 IU/ml penicillin, 100 μg/ml streptomycin (Gibco Invitrogen Corporation, California USA), 50 μM β-mercaptoethanol (KEBO-lab, Spånga, Sweden), and 10% heat inactivated fetal calf serum (HyClone SH30071.03, Thermo Scientific, Waltham, MA, USA) and kept on ice until processing. The spleens were processed individually by gentle mashing with a plunger of a 5 ml syringe in 2 ml complete medium on a 70 μm cell strainer on a Petri dish. After washing the plunger and the cell strainer with 3 ml additional medium, the cell suspension was transferred to a Falcon tube and centrifuged for 10 min at 300 g at room temperature. The supernatant was discarded and the cell pellet was resuspended in 1 ml ACK lysis buffer for 1 min (0,15 M NH_4_Cl, 10 mM KHCO_3_, 0,1 mM Na_2_EDTA, pH adjusted to 7.4). Following red blood cell lysis, the remaining cells were washed once with complete medium at 300 g for 10 min and kept on ice until further processing. The cells were counted with trypan blue exclusion and the cell viability was 87.4 ± 3.7%. (n = 36). 200 μl of cell suspension (2 × 10^5^ viable cells)/well was placed in a round bottom 96 well plate in triplicates with or without stimuli; Concanavalin A (ConA) 1 μg/ml or *T. gondii* antigen 1 or 10 μg/ml made in house [35]. After 48 h or 72 h, 1 μCi ^3^[H]-thymidine (Amersham, Buckinghamshire, UK) was added to each well for additional 18 h where after the thymidine incorporation was determined by scintillation counting. Results are expressed as mean counts per minute (cpm) ± SD.

### IFN-γ ELISA

IFN-γ levels were determined in cell culture supernatants from the splenocyte proliferation study using a mouse IFN-γ ELISA kit from MabTech (Nacka Strand, Sweden) according to manufacturer’s instructions. Results are presented as mean pg/mL ± SD of triplicates.

### Statistical analysis

Differences among the treatment groups were assessed by oneway or two-way analysis of variance (ANOVA). Significant ANOVAs were further analysed using Bonferroni, Tukeys pairwise comparison, Dunn’s post hoc test or Kruskal-Wallis test using Gaussian approximation. A p-value of less than 0.05 was considered significant. Statistical analysis and graphs were performed in Graph Pad Prism (version 5.0 GraphPad software Inc., San Diego, CA, USA) and Minitab version 15 (Minitab Inc, PA, USA).

## Abbreviations

ANOVA: Analysis of variance; BAL: Bronchoalveolar lavage; BLI: Bioluminescense imaging; CNT: Carbon nanotubes; DC: Dendritic cells; DMEM: Dulbecco’s modified eagle’s medium; ELISA: Enzyme-linked immunosorbent assay; FBS: Fetal bovine serum; HFF: Human foreskin fibroblast; IL: Interleukin; INF: Interferon; IVIS: *In vivo* imaging system spectrum; LAL: Limulus amebocyte lysate; LDH: Lactate dehydrogenase; LM: *Listeria monocytogenes*; MCP-1: Monocyte chemoattractant protein-1; MPO: Myeloperoxidase; MWCNT: Multi-walled carbon nanotubes; PBS: Phosphate buffered saline; ROI: Region of interest; SEM: Standard error of the mean; SWCNT: Single-walled carbon nanotubes; TGF: Transforming growth factor; TNF-α: Tumor necrosis factor-alpha; *T. gondii*: *Toxoplasma gondii*.

## Competing interests

The authors declare that they have no competing interest.

## Authors' contributions

LS participated in the study design, conducted the animal exposure studies, data analysis, the Luminex assay, performed differential cell counts, immunohistochemistry and lung histology; RA participated in the study design, conducted the animal exposure studies, data analysis, and performed bioluminescence imaging and plaquing assays; BA-W measured total protein and LDH levels in BAL fluid, and performed the splenocyte cultures and ELISA analysis; AM conducted the animal exposure to SWCNT; YC and MK performed immunohistology analysis on lung and spleen specimens; SKG conducted part of the Luminex assay, advised and assisted on histological methods and performance; AVT analysed data; AAS, BF, AB, AS designed the animal study; AS coordinated the study and data analysis and the first version of the manuscript together with LS and RA. All authors have contributed to draft the manuscript and have read and approved the final manuscript.

## Authors' information

Dr Bengt Fadeel, Dr Anna A Shvedova, Dr Antonio Barragan, and Dr Annika Scheynius share the senior authorship.

## Disclaimers

The findings and conclusions in this report are those of the authors and do not necessarily represent the view of the National Institute for Occupational Safety and Health.

## Supplementary Material

Additional file 1 **Increased IFN-γ production in*T. gondii*infected mice.** Single cell suspensions of spleens from mice exposed to PBS or *T. gondii* in two doses (10^3^ or 10^4^ parasites) were cultured for 48 h with or without ConA (1 μg/ml) or *T. gondii* antigen (1 or 10 μg/ml). Thereafter the culture supernatants were subjected to IFN-γ ELISA. Results are presented as mean IFN-γ pg/mL ± SD from triplicates of six-eight mice divided on two experiments. Level of significance * p < 0.05, ** p < 0.01 and *** p < 0.001 determined with two-way ANOVA, and Bonferroni’s Multiple Comparison test. (TIFF 661 kb) Click here for file

Additional file 2 **Weight loss in mice after*T. gondii*infection.** The weight of mice was measured on day 10. Data are presented as mean ± SEM. Statistical significance was determined with one-way ANOVA and Bonferroni’s Multiple Comparison test. * = p < 0.05 compared to PBS controls, *f* = p < 0.05 compared to SWCNT. (TIFF 814 kb) Click here for file
