# Supplementary figures and images for: Pulmonary exposure to single-walled carbon nanotubes does not affect the early immune response against Toxoplasma gondii
Source: Part Fibre Toxicol. 2012 May 23;9:16. doi: 10.1186/1743-8977-9-16 (PMC3495637; doi:10.1186/1743-8977-9-16)

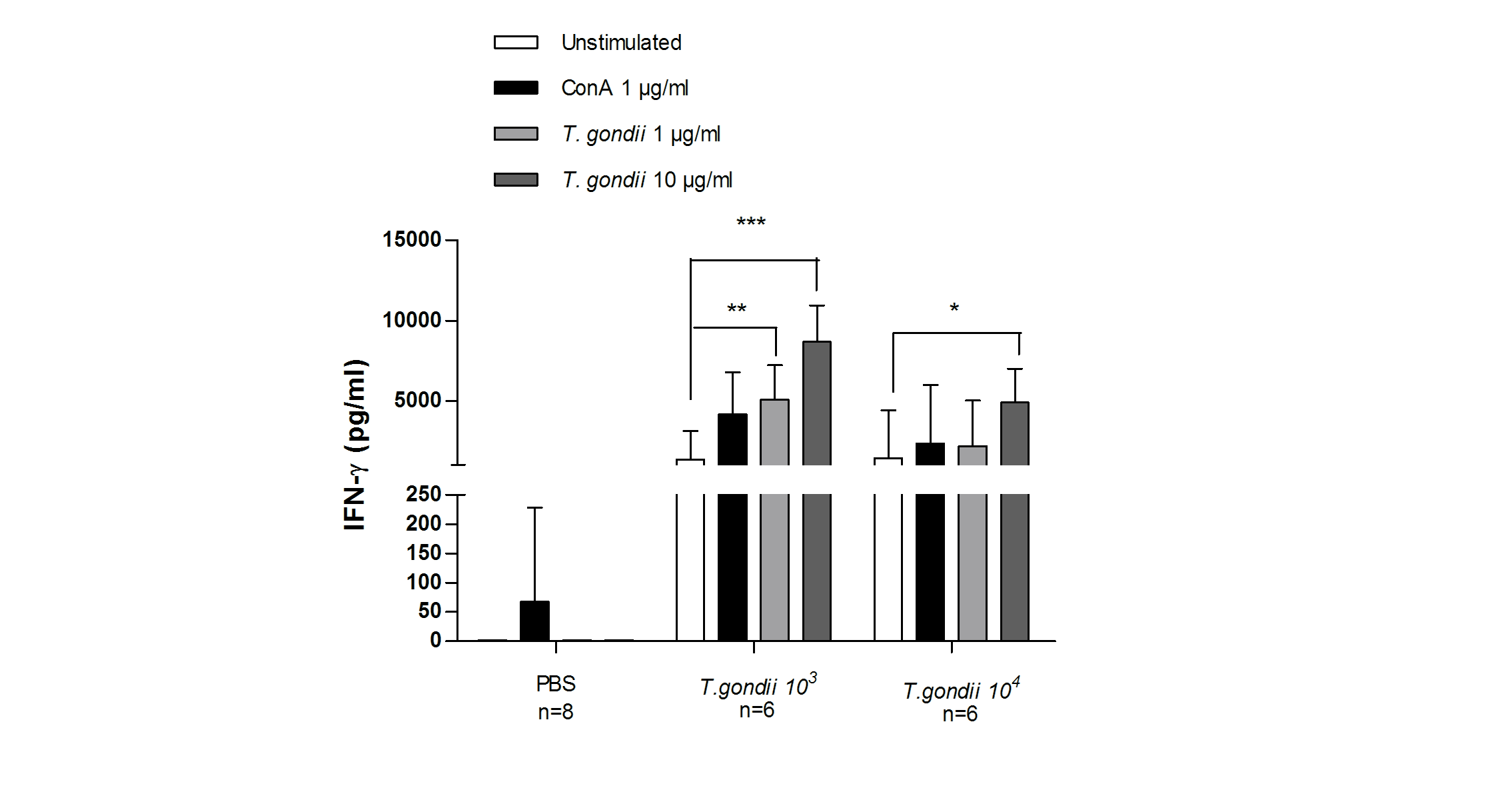

Supplement: Additional file 1 — Increased IFN-γ production inT. gondiiinfected mice. Single cell suspensions of spleens from mice exposed to PBS or T. gondii in two doses (103 or 104 parasites) were cultured for 48 h with or without ConA (1 μg/ml) or T. gondii antigen (1 or 10 μg/ml). Thereafter the culture supernatants were subjected to IFN-γ ELISA. Results are presented as mean IFN-γ pg/mL ± SD from triplicates of six-eight mice divided on two experiments. Level of significance * p < 0.05, ** p < 0.01 and *** p < 0.001 determined with two-way ANOVA, and Bonferroni’s Multiple Comparison test. (TIFF 661 kb) [file 1743-8977-9-16-S1.tiff]

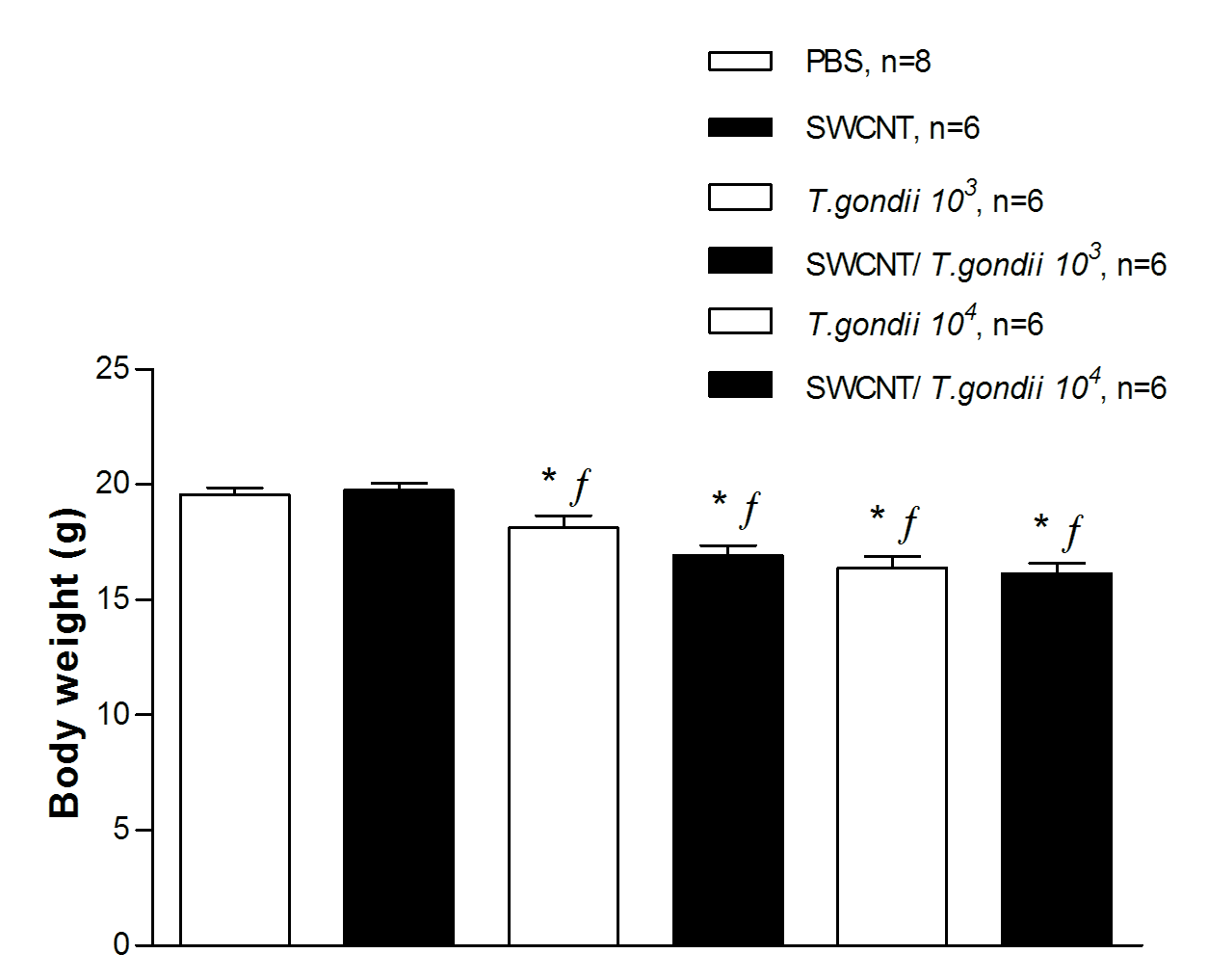

Supplement: Additional file 2 — Weight loss in mice afterT. gondiiinfection. The weight of mice was measured on day 10. Data are presented as mean ± SEM. Statistical significance was determined with one-way ANOVA and Bonferroni’s Multiple Comparison test. * = p < 0.05 compared to PBS controls, f = p < 0.05 compared to SWCNT. (TIFF 814 kb) [file 1743-8977-9-16-S2.tiff]
